# Supplementary figures and images for: Identification and validation of candidate genes dysregulated in alveolar macrophages of acute respiratory distress syndrome
Source: PeerJ. 2021 Oct 26;9:e12312. doi: 10.7717/peerj.12312 (PMC8555499; doi:10.7717/peerj.12312)

**Fig. 1**

**(A)**

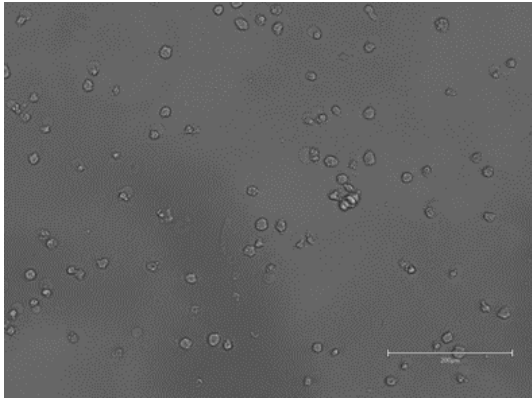

**THP-1 cells**

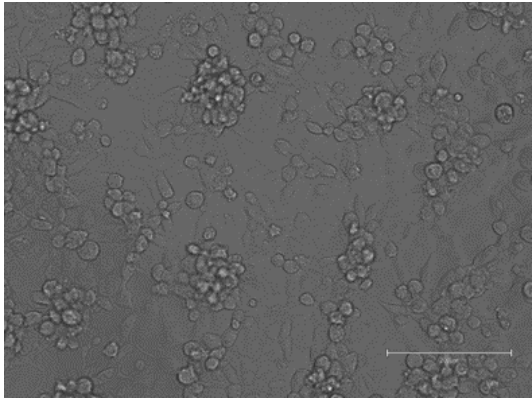

**PMA-differentiated macrophages**

**(B)**

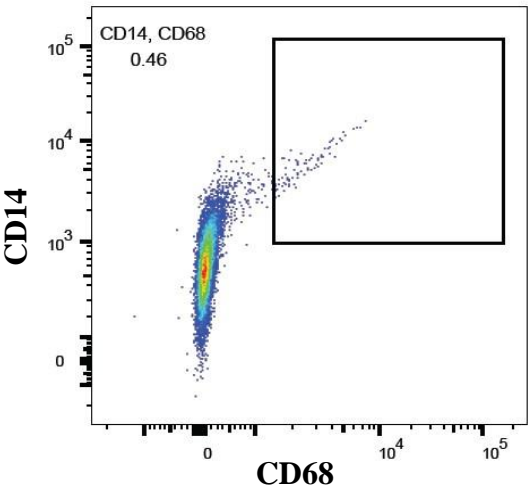

**THP-1 cells**

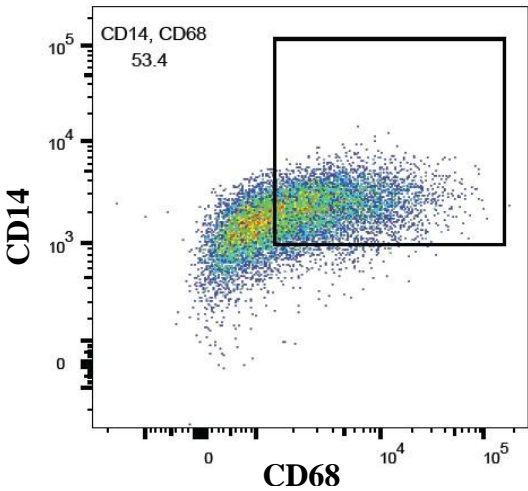

**PMA-differentiated macrophages**

**(C)**

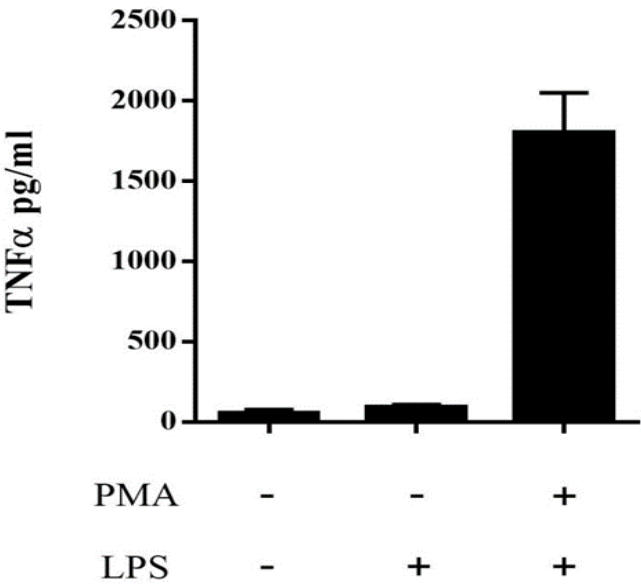

Supplement: Supplemental Information 1 — (A) Phenotypes of PMA-differentiated macrophages and non-differentiated THP-1 cells were observed by phase-contrast microscopy. (B) Surface marker CD14(monocyte) and CD68(macrophage) were detected by flow cytometry analysis. (C) The TNF α release in response to LPS was evaluated by ELISA assay. [file peerj-09-12312-s001.pdf]

**Fig. 2**

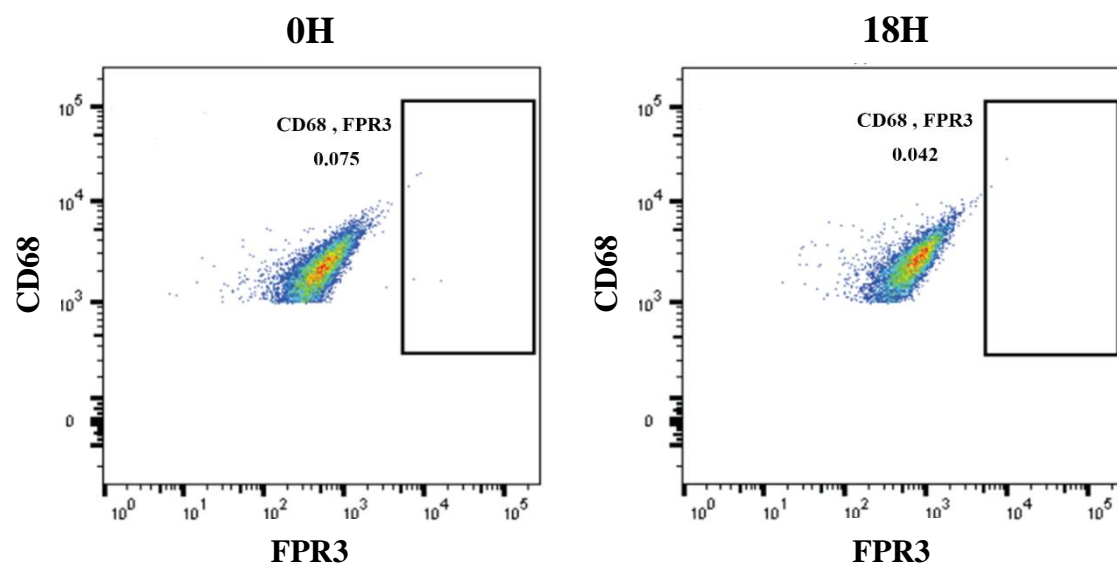

Supplement: Supplemental Information 2 [file peerj-09-12312-s002.pdf]

**Fig.3**

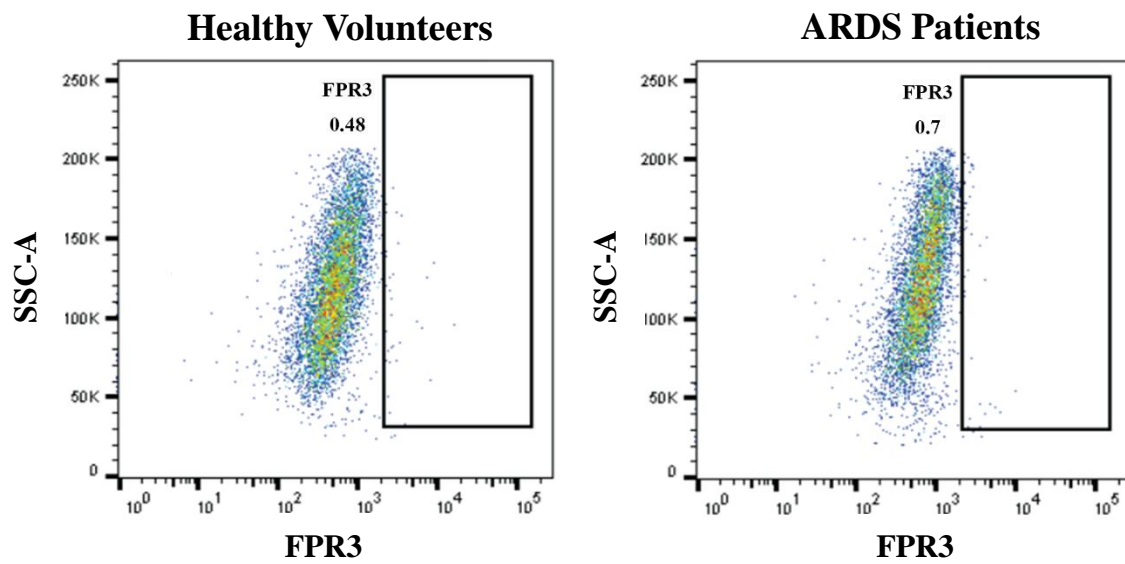

Supplement: Supplemental Information 3 [file peerj-09-12312-s003.pdf]

**Fig.4**

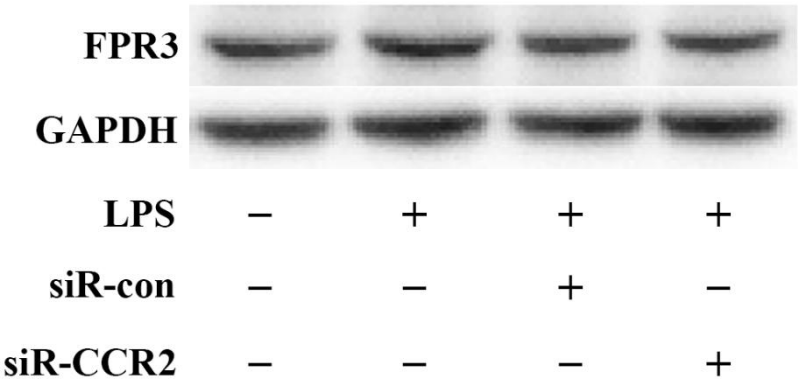

Supplement: Supplemental Information 4 [file peerj-09-12312-s004.pdf]

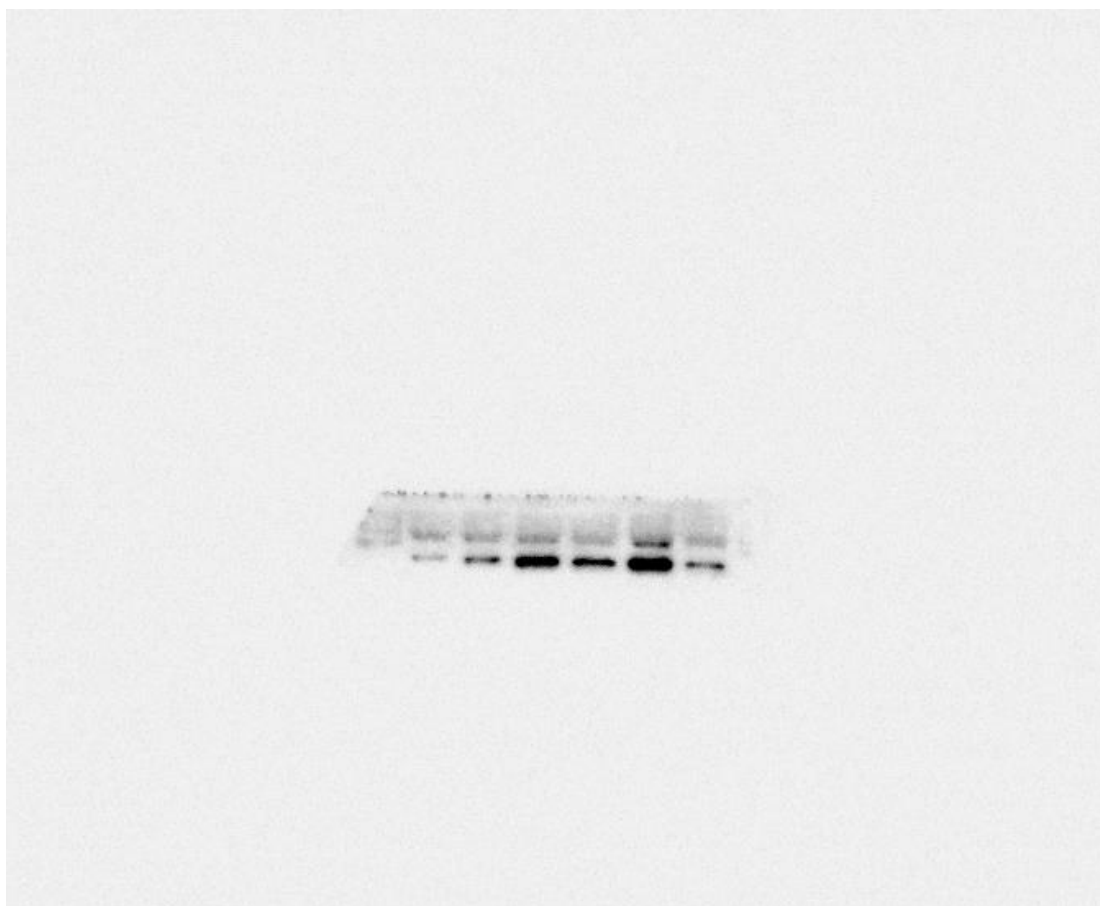

LPS-CCR2

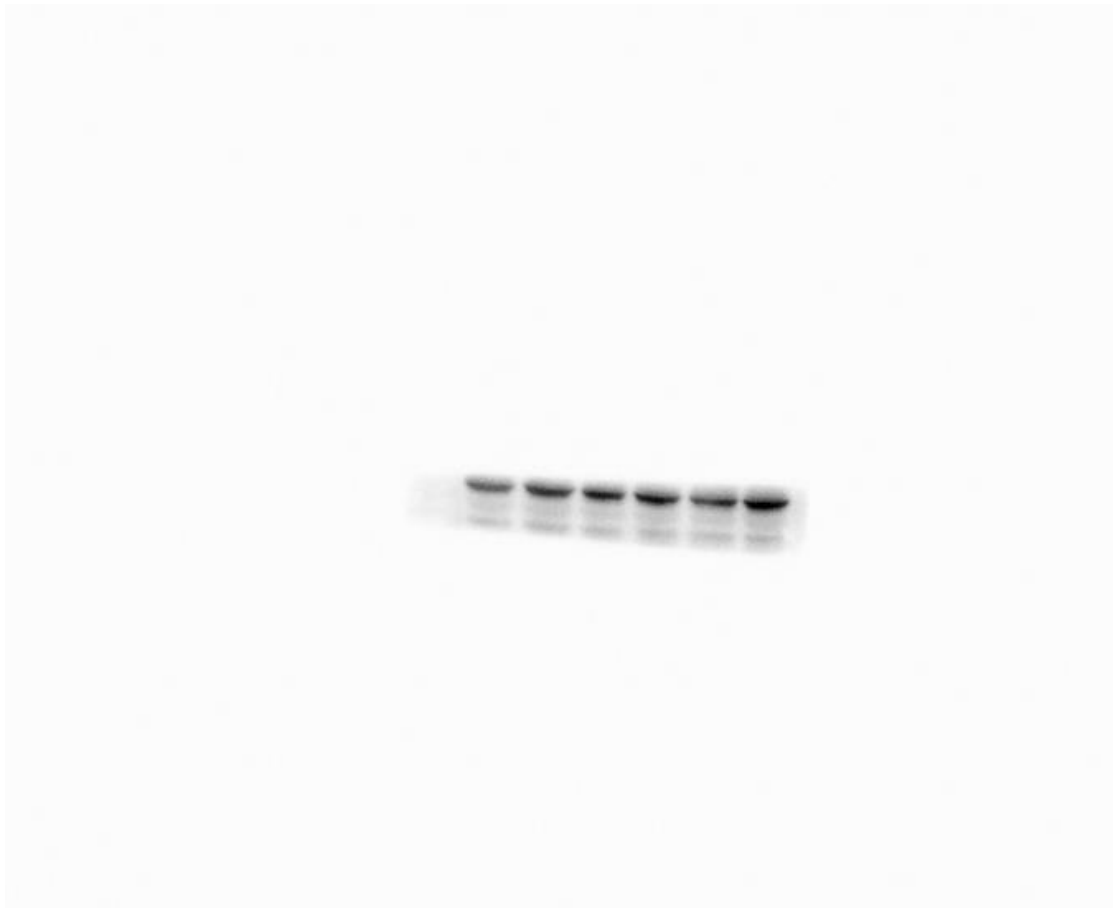

LPS-FPR3

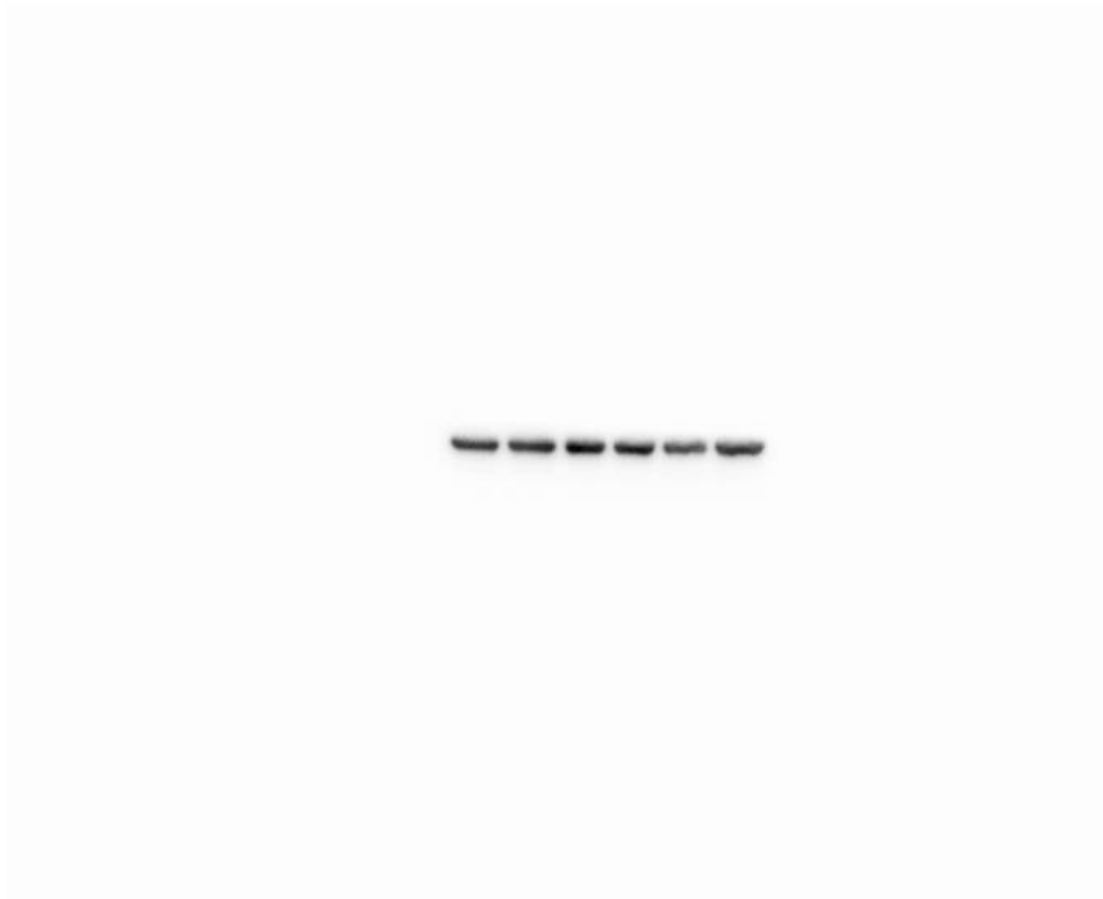

LPS-GAPDH

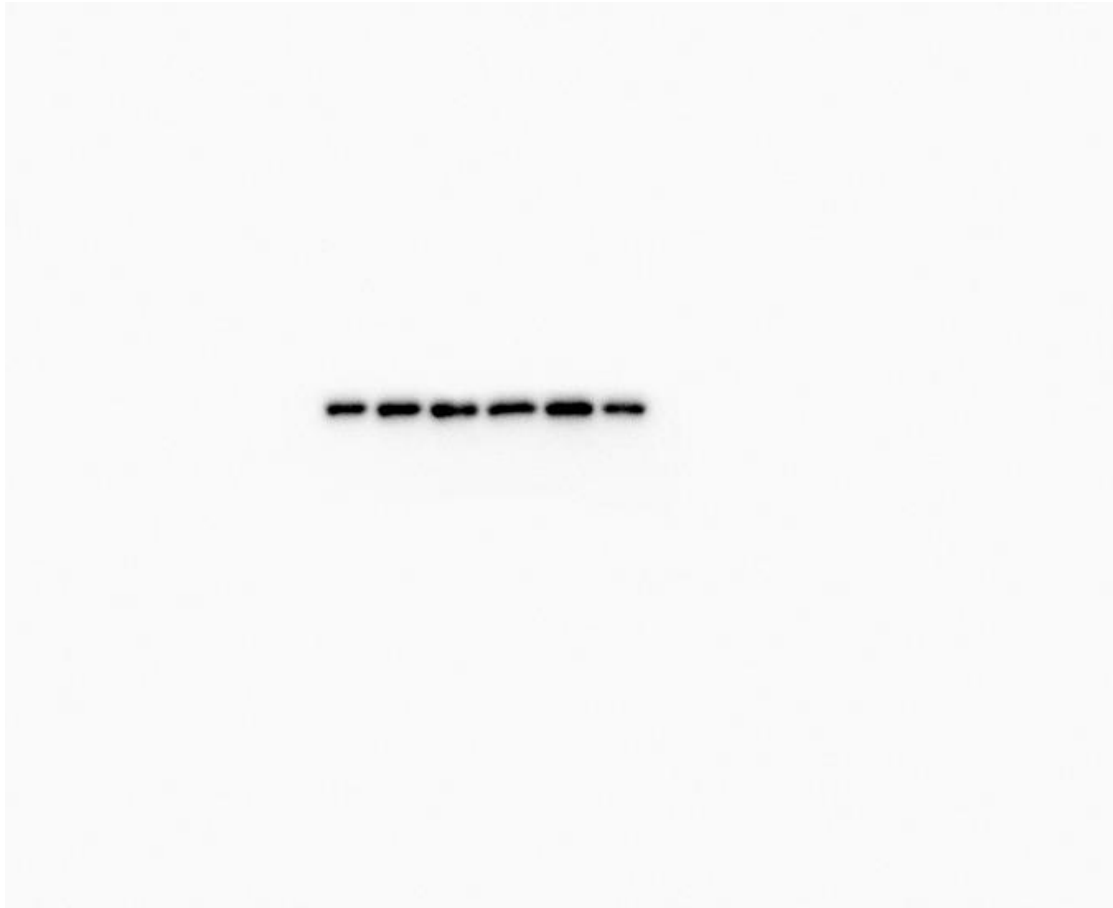

LPS-GAPDH2

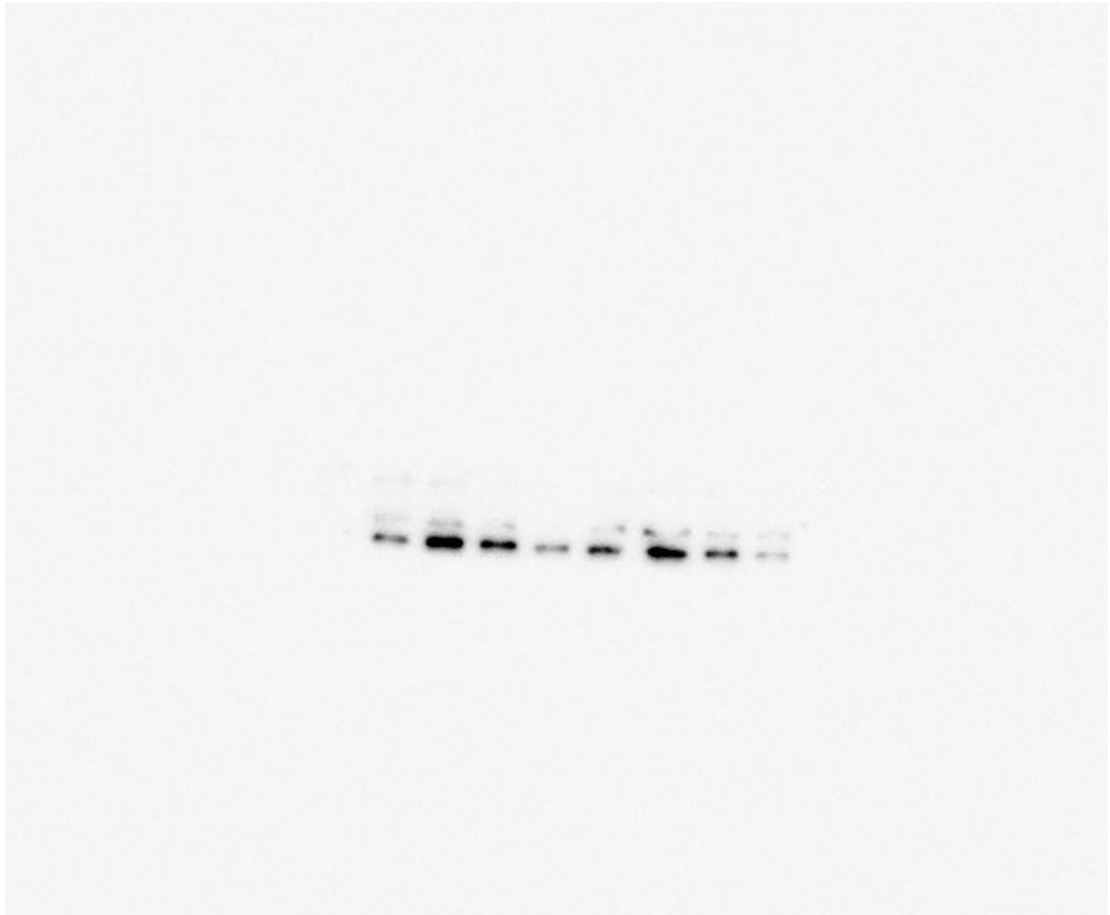

siRNA-FPR3—CCR2

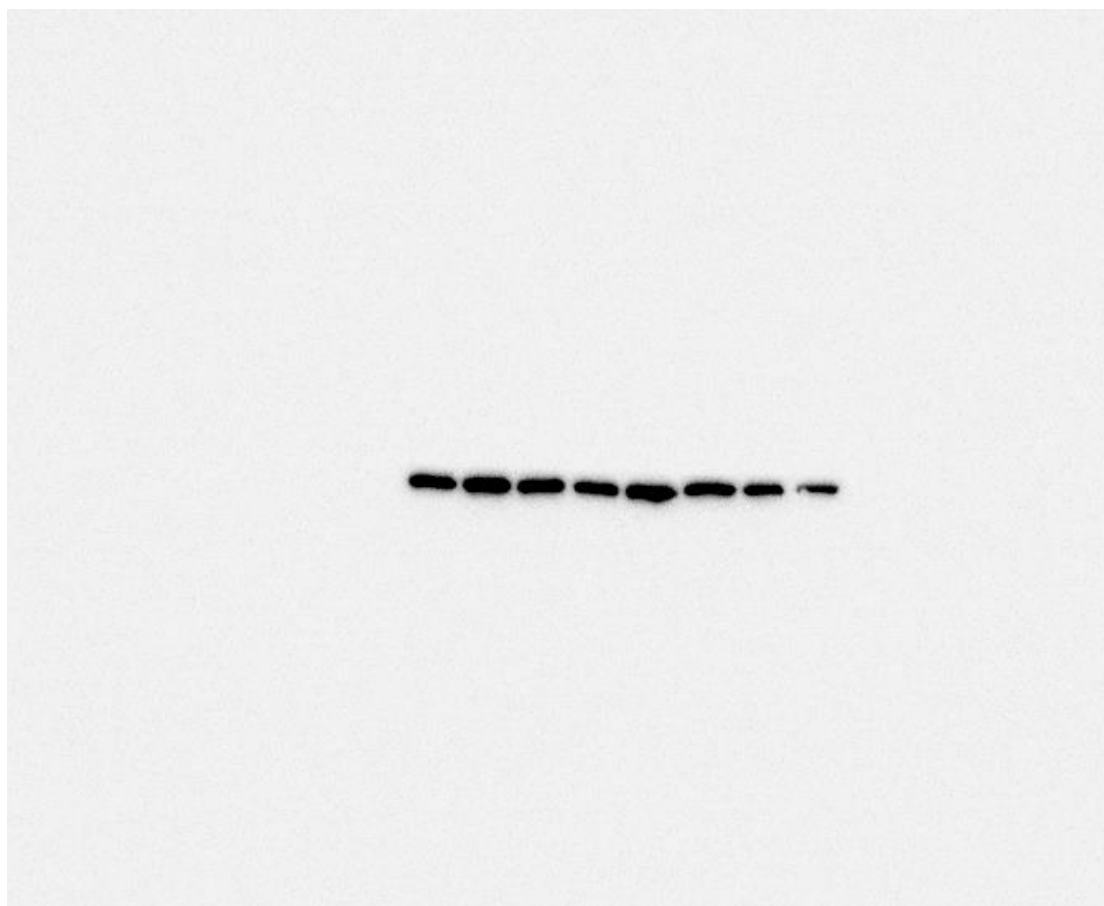

siRNA-FPR3—GAPDH

Supplement: Supplemental Information 6 [file peerj-09-12312-s006.pdf]

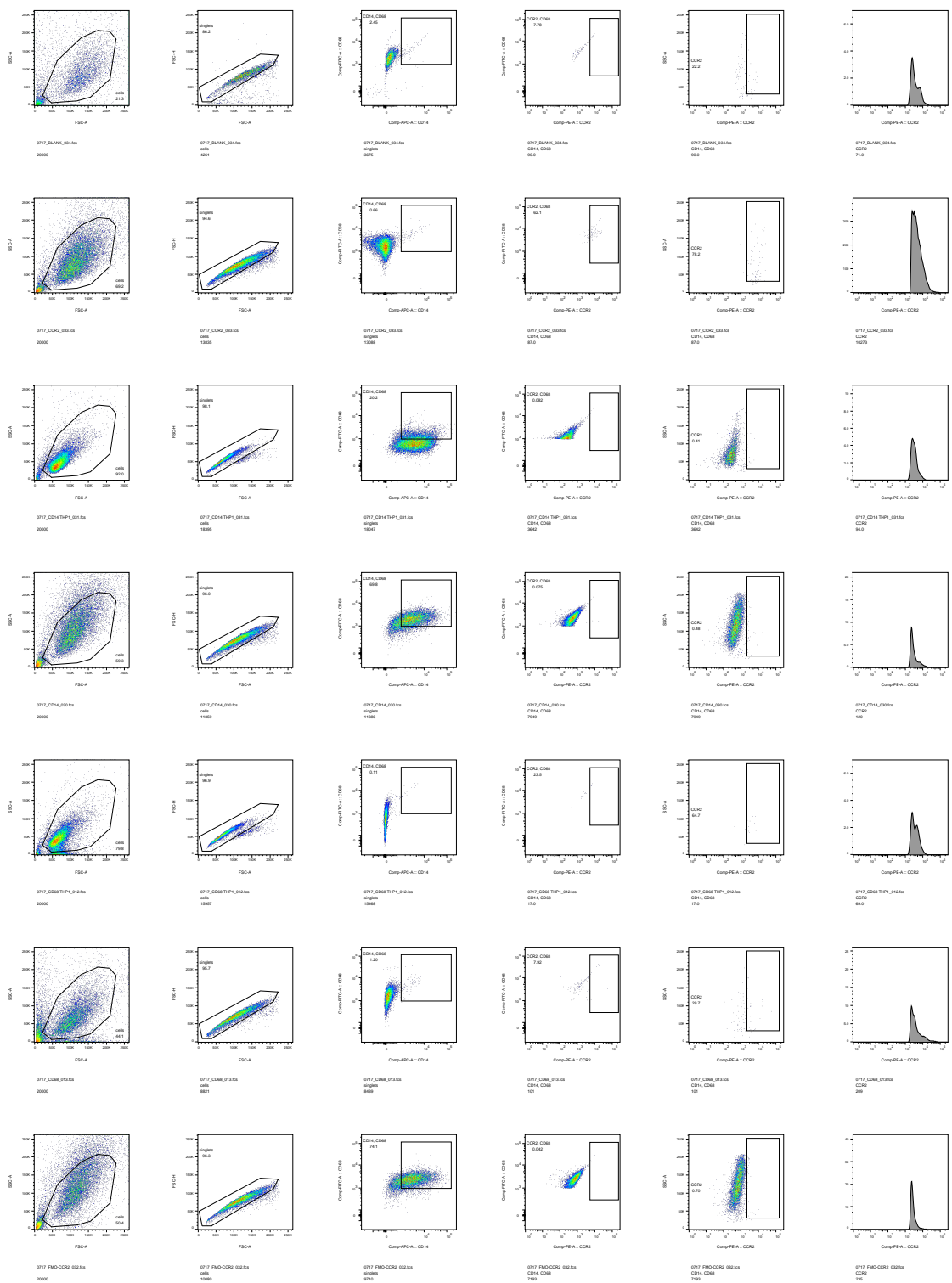

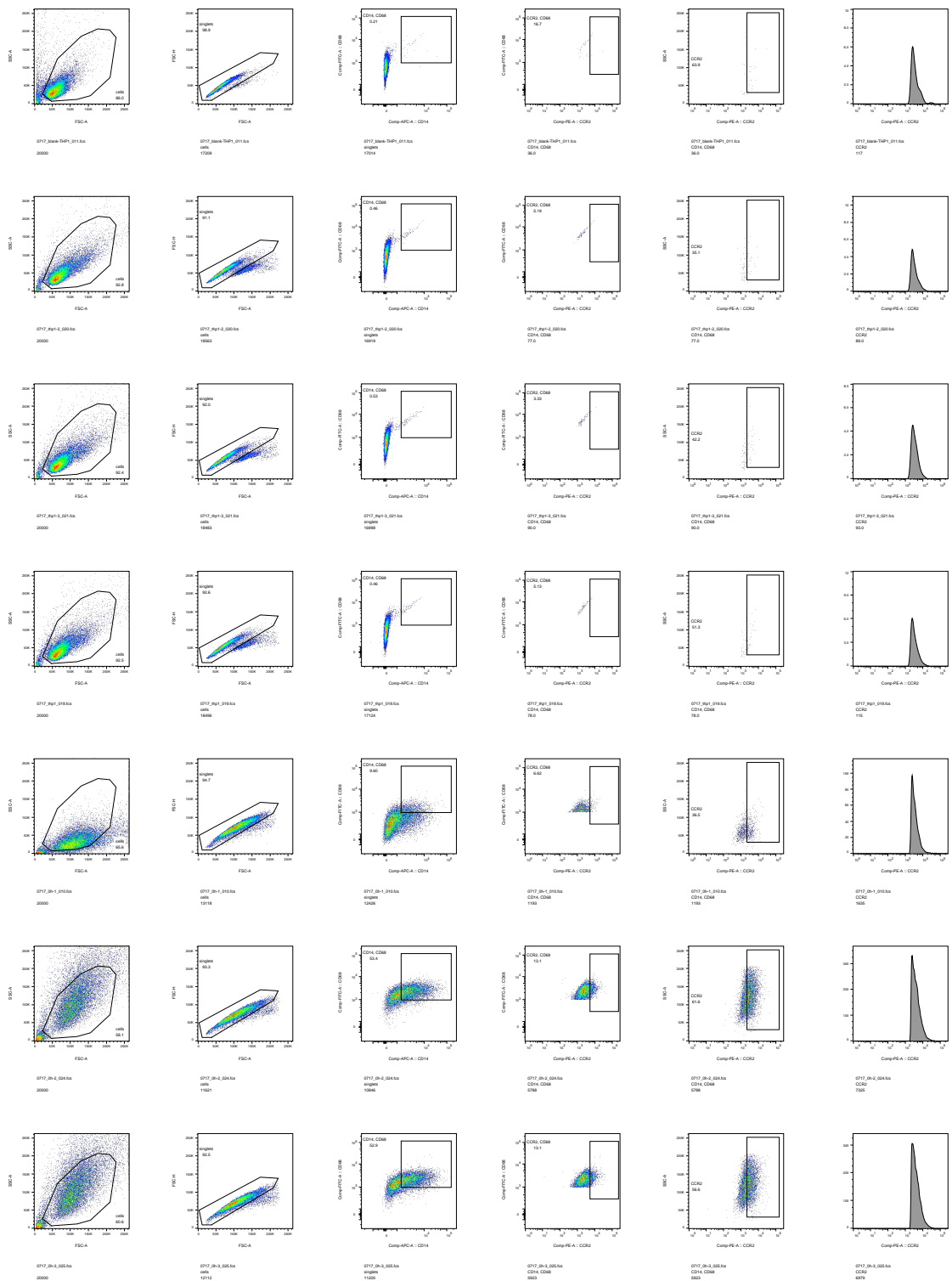

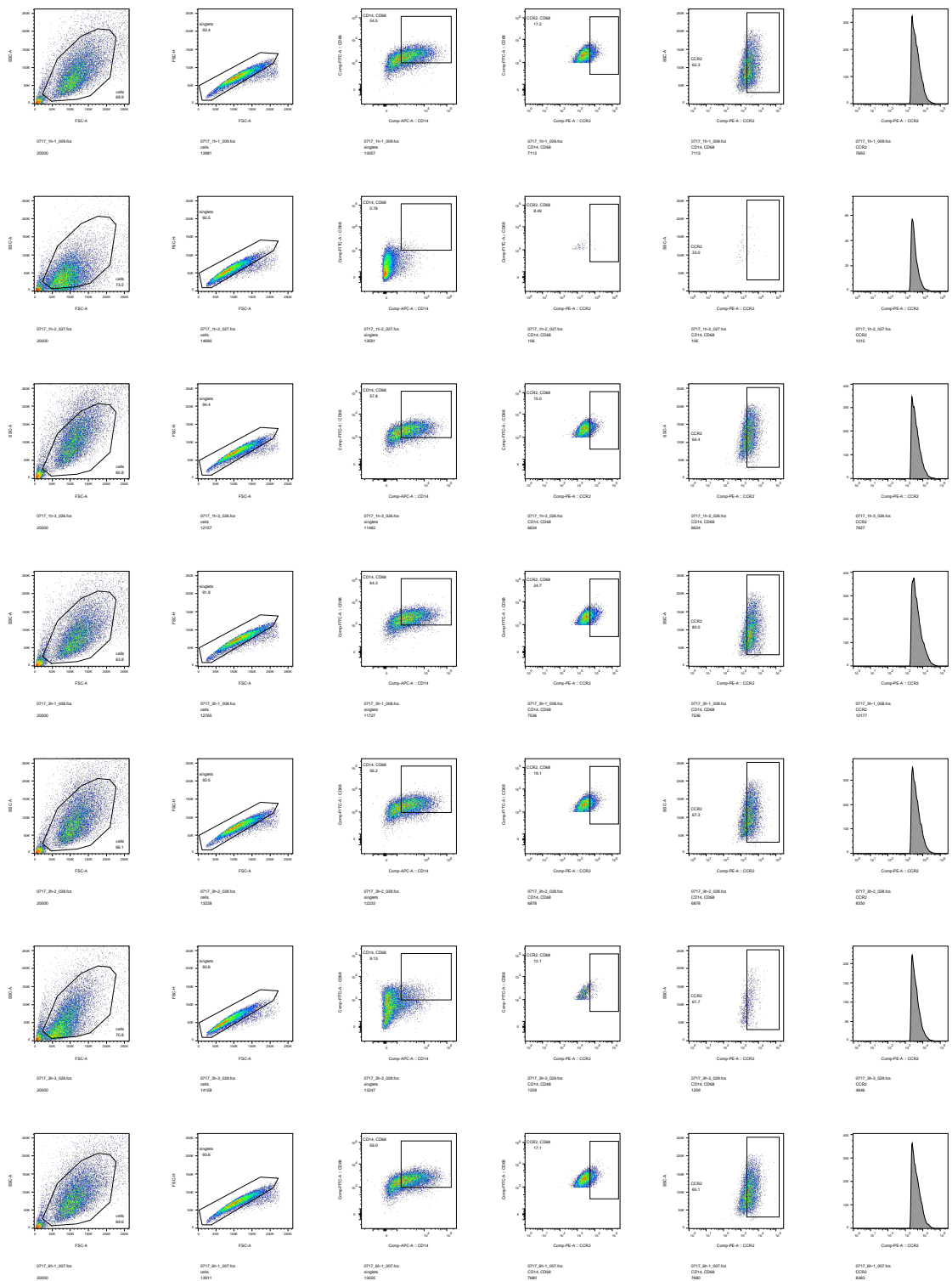

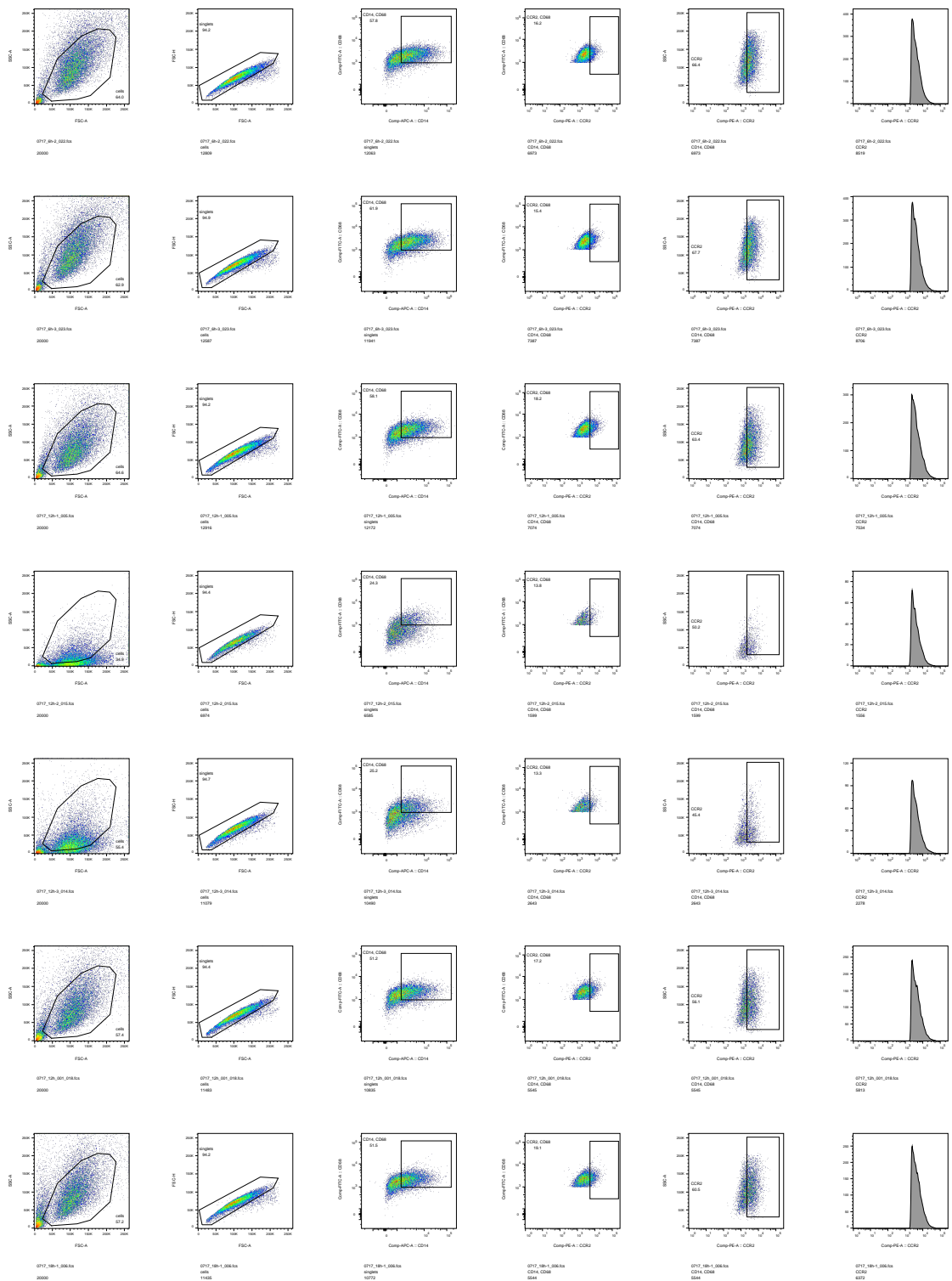

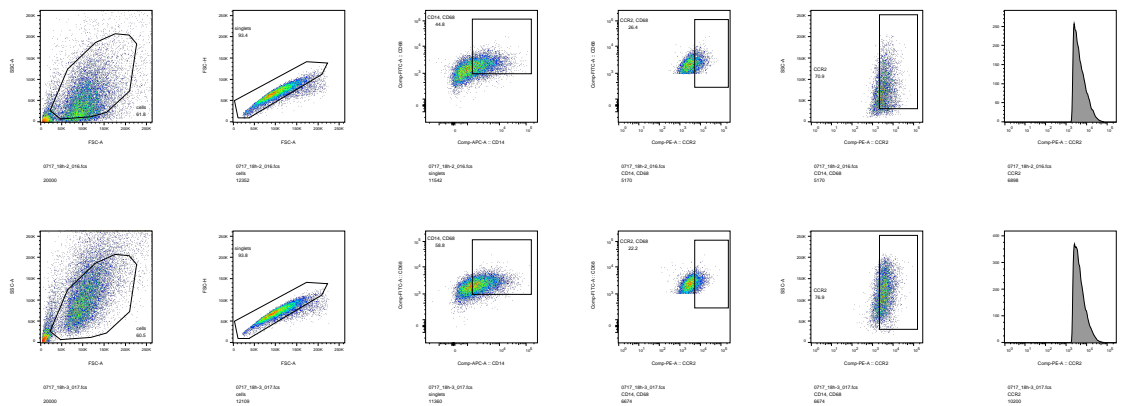

Supplement: Supplemental Information 9 [file peerj-09-12312-s009.pdf]
