# Supplementary material for: Identification and validation of candidate genes dysregulated in alveolar macrophages of acute respiratory distress syndrome
Source: PeerJ. 2021 Oct 26;9:e12312. doi: 10.7717/peerj.12312 (PMC8555499; doi:10.7717/peerj.12312)
Supplement: Supplemental Information 5 — The primer sequences of 6 hub genes including FPR3, CXCL16, CCL13, CX3CR1, PLAU and CCR2 are shown. [file peerj-09-12312-s005.pdf]

**Table1**

| Primers | Sequences |
|---|---|
| **FPR3-F** | GACTGATTCGCTCTTTGCCCAC |
| **FPR3-R** | TCTCCTCAGGAGGTGAAGCAGA |
| **CXCL16-F** | CCTATGTGCTGTGCAAGAGGAG |
| **CXCL16-R** | CTGGGCAACATAGAGTCCGTCT |
| **CCL13-F** | GATCTCCTTGCAGAGGCTGAAG |
| **CCL13-R** | TCTGGACCCACTTCTCCTTTGG |
| **CX3CR1-F** | CACAAAGGAGCAGGCATGGAAG |
| **CX3CR1-R** | CAGGTTCTCTGTAGACACAAGGC |
| **PLAU-F** | GGCTTAACTCCAACACGCAAGG |
| **PLAU-R** | CCTCCTTGGAACGGATCTTCAG |
| **CCR2-F** | TACGGTGCTCCCTGTCATAAA |
| **CCR2-R** | TAAGATGAGGACGACCAGCAT |
